# Data Mining of Adverse Reactions to Iodinated Contrast Media Based on a Municipal Spontaneous Reporting System in China

**DOI:** 10.1007/s11596-025-00141-0

**Published:** 2025-11-26

**Authors:** Wen-ting Zhang, Wei-jie Li, Jin-wen Zhang, Ya-min Shu, Yu-huan Leng, Ru-xue Xu, Qin Li, Qi-hao Cui, Xue-peng Gong, Dong Liu, Ying Jiang

**Affiliations:** 1 Department of Pharmacy, Tongji Hospital, Tongji Medical College, Huazhong University of Science and Technology, Wuhan, 430030 China; 2 Department of Pharmacy, The People’s Hospital of Huangpi District, Wuhan, 430300 China; 3 Department of Radiology, Tongji Hospital, Tongji Medical CollegeHuazhong University of Science and Technology, Wuhan, 430030 China; 4 Municipal Center for Adverse Drug, Reaction Monitoring of Wuhan, Wuhan, 430022 China

**Keywords:** Iodinated contrast media, Adverse drug reactions, Spontaneous reporting system

## Abstract

**Objective:**

Iodinated contrast media (ICM) are widely used in medical imaging, particularly in computed tomography (CT) and magnetic resonance imaging (MRI) examinations, to increase image quality and improve diagnostic accuracy. Despite their clinical utility, ICM are associated with various adverse drug reactions (ADRs), including allergic reactions and other systemic effects. This study aimed to evaluate ICM-related ADRs through the Chinese spontaneous reporting system (SRS) and provide reference information for clinical practice.

**Methods:**

We analyzed ADRs related to ICM on the basis of data from the SRS in Wuhan, China, from January 1, 2018, to December 31, 2023.

**Results:**

A total of 2,166 ADR reports related to four ICM (iodixanol, iohexol, ioversol, and iomeprol) were analyzed to assess the proportion and severity of adverse reactions. The results revealed that the majority of the ADRs were mild, with the most common symptoms being rash (54.76%) and itching (35.18%). Gastrointestinal and respiratory symptoms were also noted, although less frequently. Anaphylactic shock was documented in 48 patients, accounting for 9.69% of severe adverse reactions. The incidence of ADRs was greater in summer. Circulatory system diseases were the most prevalent underlying conditions in patients who experienced ADRs. Treatment primarily involved symptomatic management, including corticosteroids and antihistamines, with adrenaline administered in severe cases.

**Conclusions:**

This study highlights the importance of monitoring high-risk patients, especially elderly patients and those with preexisting conditions, and underscores the need for timely intervention in severe reactions. Future prospective studies are necessary to facilitate the selection of more appropriate ICM for individual patients.

## Introduction

Iodinated contrast media (ICM) are widely employed in medical imaging, particularly in enhanced computed tomography (CT) and magnetic resonance imaging (MRI) examinations [1]. Their use substantially enhances image contrast, thereby facilitating more precise disease diagnosis. However, despite their clinical benefits, the administration of ICM is associated with certain risks, including hypersensitivity and other adverse events [2, 3].

The Spontaneous Reporting System (SRS) serves as a pivotal pharmacovigilance instrument for collecting real-world data on adverse reactions, thereby providing essential insights into the safety profile of ICM [4]. By providing comprehensive insights into the safety profile of iodinated contrast agents, the SRS facilitates evidence-based clinical decision-making and the development of risk mitigation strategies [5]. It offers critical data support for the surveillance of adverse reactions to iodinated contrast agents [4]. The analysis of the data enables healthcare professionals to identify and manage adverse reactions more effectively, ultimately enhancing patient safety in medication use. In light of these considerations, pharmacovigilance is indispensable for ensuring the safe clinical application of ICM through the continuous monitoring of its safety profile. Moreover, research has indicated regional and population-based variations in adverse reactions to iodinated contrast agents [6]. For example, a study conducted in Chongqing, China, reported a low incidence of adverse reactions to non-ionic iodinated contrast agents, with the majority of cases being mild [7]. This finding indicates that, with suitable precautions, the utilization of these agents is generally considered relatively safe. Similarly, a multicenter study conducted in South Korea reported an overall incidence of allergic reactions to iodinated contrast agents of 0.73%, with severe reactions occurring at a rate of 0.01% [8]. The study additionally identified personal and familial histories of allergies as significant risk factors for the occurrence of these reactions.

In recent years, research on the safety of ICM in China has been limited. Existing studies often rely on small sample sizes, limiting their ability to accurately capture the patterns of contrast agent-induced adverse drug reactions (ADRs) in the Chinese population. This study undertook a retrospective analysis of ADR data related to ICM, sourced from the SRS in Wuhan, Hubei Province, spanning from January 1, 2018, to December 31, 2023. The primary aim of this investigation was to assess the safety profile of iodinated contrast agents and to offer evidence-based recommendations for reducing the clinical risks associated with their use.

## Methods

### Data Sources

The ADR monitoring data for pharmaceuticals in Wuhan were sourced from the city’s pharmacovigilance system, which consolidates information from the National Adverse Drug Reaction Monitoring SRS. The data collection period spans from January 1, 2018, to December 31, 2023. To maintain the accuracy, professionalism, and traceability of the data, this retrospective study exclusively included reports submitted by healthcare institutions that were suspected to be adverse reactions associated with ICM. Duplicate and erroneous reports were systematically excluded from the analysis.

### Report Screening and Data Organization

The ADRs of the ICM were mapped to their respective “ADR-system/organ” categories on the basis of the World Health Organization Adverse Reaction Terminology (WHOART). The results of the causality assessment for each ADR by the WHO criteria were recorded in the database by the reporter and were assessed by the Municipal Center for ADR Monitoring of Wuhan. The WHO causality assessment tool was employed to evaluate the reported ADRs and ascertain the relationship between the suspected pharmaceutical agent and the observed adverse effects. Six categories were used to evaluate the causal relationships: certain, probable, possible, unlikely, conditional or unclassified, and unclassifiable. We chose only three categories as causal ADRs: certain, probable, and possible.

The serious ADRs were classified as follows: death; life-threatening events; causing or prolonged hospitalization; causing persistent significant disability or incapacity; resulting in a congenital anomaly or birth defect; and other medical conditions.

A total of 2,166 ADR cases related to four iodinated contrast agents—iodixanol, iohexol, ioversol, and iomeprol—were identified through comparison with the drug labels. All ADR data were subsequently processed for analysis.

### Statistical Analysis

Data were analyzed using Microsoft Excel 2019 and SPSS version 22.0. The χ^2^ test was used for comparisons, and a *P* value of < 0.05 was considered statistically significant.

## Results

### Demographic Characteristics

In an analysis of 2,166 reports concerning ADRs associated with iodinated contrast agents, 1,165 cases involved male patients, accounting for 53.79% of the total, whereas 1,001 cases involved female patients, accounting for 46.21%. Among these individuals, 994 (45.89%) were over the age of 60, whereas 1,170 (54.02%) were aged 60 years or younger. With respect to the professional background of the reporting individuals, physicians submitted 946 reports (43.67%), pharmacists contributed 686 reports (31.67%), and nurses accounted for 534 reports (24.65%) (Table 1).

**Table 1:** Demographic characteristics of respondents

Characteristics	Iodixanol (n = 1,122)	Iohexol (n = 751)	Ioversol (n = 163)	Iomeprol (n = 130)
Sex, n (%)				
Female	505 (45.01%)	358 (47.67%)	74 (45.4%)	64 (49.23%)
Male	617 (54.99%)	393 (52.33%)	89 (54.6%)	66 (50.77%)
Age (years), n (%)				
≤60	554 (49.38%)	456 (60.72%)	87 (53.37%)	73 (56.15%)
>60	566 (50.45%)	295 (39.28%)	76 (46.63%)	57 (43.85%)
Unknown	2 (0.18%)	0 (0%)	0 (0%)	0 (0%)
Median (IQR)	61 (52–69)	57 (49–65)	59 (51–67)	59 (48.25–66.75)
Weight (kg), n (%)				
≤60	345 (30.75%)	218 (29.03%)	58 (35.58%)	48 (36.92%)
>60	420 (37.43%)	266 (35.42%)	76 (46.63%)	60 (46.15%)
Unknown	357 (31.82%)	267 (35.55%)	29 (17.79%)	22 (16.92%)
Median (IQR)	63 (55.5–70)	62.55 (55–70)	64 (55–70)	63.5 (53.88–72.63)
Reported area, n (%)				
Qingshan District	210 (18.72%)	71 (9.45%)	6 (3.68%)	0 (0%)
Jianghan District	207 (18.45%)	290 (38.62%)	6 (3.68%)	61 (46.92%)
Wuchang District	164 (14.62%)	152 (20.24%)	34 (20.86%)	0 (0%)
Jiang’an District	129 (11.5%)	53 (7.06%)	25 (15.34%)	24 (18.46%)
Qiaokou District	127 (11.32%)	57 (7.59%)	25 (15.34%)	6 (4.62%)
Reporters, n (%)				
Doctor	559 (49.82%)	264 (35.15%)	85 (52.15%)	38 (29.23%)
Pharmacist	467 (41.62%)	135 (17.98%)	43 (26.38%)	41 (31.54%)
Nurse	96 (8.56%)	352 (46.87%)	35 (21.47%)	51 (39.23%)

IQR, interquartile range

**Fig. 1:**
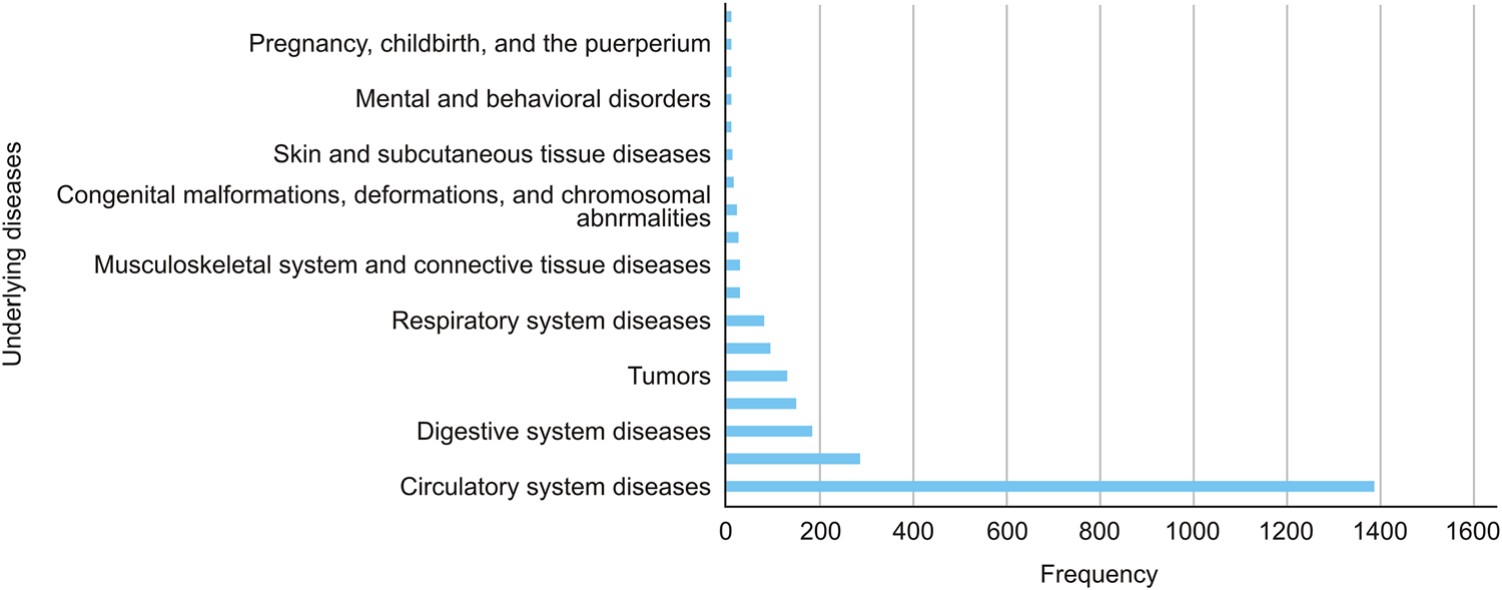
Underlying diseases of patients

A comprehensive analysis of the patients’ underlying conditions was performed, resulting in the identification and classification of 28 distinct disease systems. Subsequent statistical analysis and frequency ranking revealed that circulatory system diseases were the most prevalent, with 1,390 cases. Among these, coronary heart disease, hypertension, cerebral infarction, and coronary atherosclerotic heart disease were the most frequently observed. Furthermore, diseases of the digestive system (181 cases), endocrine and metabolic disorders (147 cases), and neoplasms (128 cases) were ranked third to fifth, respectively (Fig. 1).

### Comparison of ADR Characteristics Among Different ICM

Among the 2,166 ADR reports, 1,122 cases (51.8%) were associated with iodixanol, 751 cases (34.7%) with iohexol, 163 cases (7.5%) with ioversol, and 130 cases (6.0%) with iomeprol. With respect to ADR outcomes, 2,122 patients (97.97%) achieved recovery or improvement. Among the four ICM reported, Ioversol had the highest proportion of severe adverse reactions, with 30.67% of the reported adverse reactions classified as severe. In comparison, the proportions of severe adverse reactions to the other three contrast agents—iodixanol, iohexol, and iomeprol—were 22.99%, 20.64%, and 18.46%, respectively (Table 1). Notably, two fatalities associated with ADRs involving iohexol were reported. The direct causes of death were identified as anaphylactic shock and laryngeal edema.

The incidence of adverse reactions to various iodine contrast agents varies seasonally, with a significantly greater number of adverse reactions occurring in summer than in other seasons. Specifically, 678 cases were reported during the summer, representing 31.3% of the total cases. In contrast, the incidence of adverse reactions was lowest in winter (Table 2). Notably, 1,541 ADRs, accounting for 71.14% of the total, occurred on the same day the ICM was administered. Importantly, 38.24% and 27.69% of the reactions associated with iodixanol and iomeprol, respectively, manifested more than 24 h after ICM administration.

**Table 2:** Comparison of ADR Characteristics Among Different ICM

Characteristics	Iodixanol, n = 1,122	Iohexol, n = 751	Ioversol, n = 163	Iomeprol, n = 130
Severity, n (%)				
Nonserious	864 (77.01%)	596 (79.36%)	113 (69.33%)	106 (81.54%)
Serious	258 (22.99%)	155 (20.64%)	50 (30.67%)	24 (18.46%)
Outcomes				
Recovered/improved	1097 (97.77%)	735 (97.87%)	161 (98.77%)	129 (99.23%)
Others	25 (2.23%)	16 (2.13%)	2 (1.23%)	1 (0.77%)
Time-to-onset (days)				
0	693 (61.76%)	630 (83.89%)	124 (76.07%)	94 72.31%)
>0	429 (38.24%)	121 (16.11%)	39 (23.93%)	36 (27.69%)
Median (IQR)	0 (0–1)	0 (0–0)	0 (0–0)	0 (0–1)
Season, n (%)				
Spring	303	201	47	41
Summer	344	244	50	40
Autumn	309	187	46	21
Winter	166	119	20	28

IQR, interquartile range

### Clinical Manifestations and Distribution of ADRs to ICM

The most prevalent adverse reaction was rash, accounting for 54.76% of all adverse reactions, as detailed in Table 3. This was followed by itching, reported in 762 cases (35.18%). Gastrointestinal-related adverse reactions predominantly included nausea, which occurred in 125 patients (5.77%), and vomiting, which was observed in 105 patients (4.85%). Respiratory-related adverse reactions included chest tightness in 57 patients (2.63%), dyspnea in 28 patients (1.29%), and throat tightness in 15 patients (0.69%). Among the severe adverse reactions, rash was the most common, accounting for 41.65% of all adverse reactions (Table 3). It was succeeded by itching, reported in 143 cases (29.48%). Furthermore, anaphylactic shock was documented in 48 patients, accounting for 9.69% of the severe adverse reactions. Chills were noted in 32 patients. With respect to severe respiratory-related adverse reactions, chest tightness was observed in 30 patients, dyspnea in 24 patients, and throat tightness in 14 patients, accounting for 14.04% of all patients.

**Table 3:** Frequencies of potential ADR symptoms reported by patients

All adverse reaction	Case number (n)	Proportion (%)	Severe ADRs	Case number (n)	Proportion (%)
Rash	1,186	54.76	Rash	202	41.65
Itching	762	35.18	Itching	143	29.48
Nausea	125	5.77	Anaphylactic shock	47	9.69
Vomiting	105	4.85	Chills	32	6.60
Chills	63	2.91	Chest tightness	30	6.19
Allergic reaction	74	3.42	Dyspnea	24	4.95
Chest tightness	57	2.63	Palpitations	20	4.12
Facial edema	54	2.49	Nausea	17	3.51
Anaphylactic shock	49	2.26	Erythema	17	3.51
Flushing	48	2.22	Allergic dermatitis	16	3.30
Erythema	42	1.94	Facial edema	16	3.30
Dizziness	40	1.85	Allergic reaction	14	2.89
Palpitations	39	1.80	Throat tightness	14	2.89
Periorbital edema	38	1.75	Dizziness	13	2.68
Urticaria	37	1.71	Flushing	12	2.47
Allergic dermatitis	32	1.48	Fatigue	12	2.47
Wheals	30	1.39	Elevated blood pressure	12	2.47
Erythematous rash	50	2.31	Wheals	11	2.27
Dyspnea	28	1.29	Vomiting	11	2.27
Local skin reaction	28	1.29	Fever	10	2.06
Pruritus	26	1.20			
Fever	21	0.97			
Papules	19	0.88			
Edema	19	0.88			
Facial swelling	18	0.83			
Fatigue	17	0.78			
Lightheadedness	16	0.74			
Throat tightness	15	0.69			
Palpitations	14	0.65			
Skin flushing	13	0.60			
Elevated blood pressure	13	0.60			
Drug-induced dermatitis	13	0.60			
Skin erythema and edema	10	0.46			
Pruritic rash	10	0.46			
Headache	10	0.46			

**Fig. 2:**
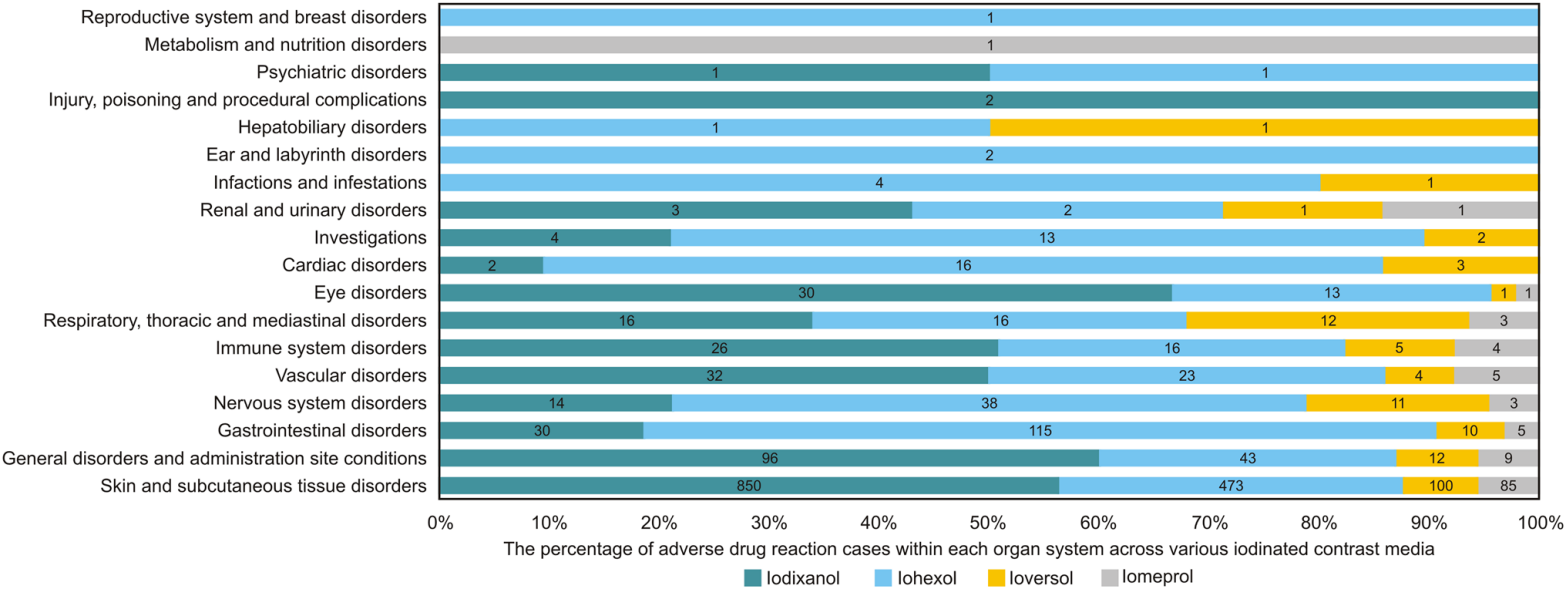
Number of ADR symptoms affecting each organ system

A total of 2,166 ADRs were categorized across 17 distinct systems/organs. The predominant category was skin and subcutaneous tissue disorders, accounting for 1,508 cases (69.62%). This was followed by general disorders, administration site conditions, and gastrointestinal disorders, each constituting 7.39% of the total ADRs. Statistical analysis of the system organ classes involved in ADRs associated with the four iodinated contrast agents revealed distinct distribution patterns. Notably, among the ADRs associated with iodixanol, a significantly greater proportion (75.76%) pertained to skin and subcutaneous tissue disorders than did those associated with iohexol, iopromide, and iomeprol, with proportions ranging from 61.35 to 65.38%. Conversely, ADRs related to iohexol were predominantly associated with gastrointestinal disorders, accounting for 15.31%, a figure significantly higher than those reported for the other three agents, which ranged from 2.67 to 6.13%. Furthermore, cardiac disorders were more frequently reported with iohexol (2.13%) and iopromide (1.84%), whereas the incidence was substantially lower for iodixanol (0.18%), and no cardiac-related ADRs were reported for iomeprol (Fig. 2).

A total of 128 novel severe ADRs were identified in the study. Notably, 30 instances of chest discomfort were documented, with 10 cases linked to iodixanol, 14 to iohexol, 3 to iopromide, and 3 to iomeprol. Furthermore, liver-related ADRs, including one case of hepatocellular damage associated with iodixanol and one case each of liver function abnormality linked to iohexol and iopromide, were detected. Additionally, among the severe ADRs related to iodixanol, 6 cases of palpitations, 5 cases of eyelid edema, 4 cases of periorbital edema, 2 cases of exfoliative dermatitis (drug eruption), and 2 cases of generalized edema have been reported. For iohexol, there were 4 patients with chills and 2 patients with dyspnea. Two cases of lip swelling, 2 cases of shortness of breath, and 2 cases of hoarseness were reported.

### Treatment of ICM-Related ADRs

In managing adverse reactions associated with ICM, symptomatic treatment is predominantly employed. Specifically, corticosteroid therapy was administered in 1,223 cases, with dexamethasone injection being the primary agent used in 1,005 instances. Antihistamine treatment was provided in 1,116 cases, commonly utilizing loratadine tablets, promethazine injection, and diphenhydramine injection. Additional therapeutic interventions included the application of calamine lotion in 94 patients, fish lotion in 20 patients, and adrenaline injection in 18 patients.

## Discussion

This study offers a comprehensive analysis of the demographic characteristics, clinical manifestations, and treatment outcomes of patients who experienced ADRs related to ICM. The findings underscore several significant aspects concerning the nature, distribution, and management of these adverse reactions.

A total of 2,166 reports of ICM-related ADRs were collected, comprising 1,165 male cases (53.79%) and 1001 female cases (46.21%), indicating a marginally higher incidence of ADRs in males; however, this difference lacked statistical significance[9]. In terms of age distribution, 45.89% of patients were over 60 years old, whereas 54.02% were 60 years or younger. This aligns with previous studies suggesting that the use of iodinated contrast agents is more prevalent among elderly patients, likely due to their increased frequency of undergoing imaging examinations[10].

This study revealed seasonal variation in the incidence of ICM-related ADRs, with the highest incidence occurring in summer, accounting for 31.3% of the total cases, and the lowest incidence observed in winter. These findings align with those of Zeng et al., who also reported an elevated risk of acute adverse reactions during the summer and autumn seasons[11]. The climate in Wuhan is characterized by elevated temperatures during the summer and autumn seasons. This increase in temperature heightens the risk of dehydration, which can lead to hemoconcentration and consequently increase the concentration of ICM, thereby increasing the risk of adverse reactions. Additionally, the summer season is associated with increased levels of environmental allergens, such as pollen and dust mites, which may exacerbate allergic reactions. However, these hypotheses require further validation through experimental data.

In terms of ADR outcomes, 97.97% of patients ultimately recovered or improved, with two fatalities reported, both of which were linked to anaphylactic shock and laryngeal edema associated with iohexol. Although the majority of adverse reactions related to ICM are mild, severe reactions, such as anaphylactic shock, still pose a risk of fatality. Prompt recognition and timely intervention in instances of anaphylactic shock are particularly critical. Patients at high risk, such as those with a history of allergies or asthma, should undergo a thorough evaluation before the administration of ICM and the implementation of preventive measures. Additionally, for patients with known allergic reactions, premedication with agents such as antihistamines and corticosteroids may be effective in mitigating the occurrence of severe reactions[12].

With respect to underlying health conditions, diseases of the circulatory system, such as coronary heart disease, hypertension, and cerebral infarction, represented the largest proportion of ADR cases. These findings suggest that patients with cardiovascular diseases may be more susceptible to adverse reactions to ICM. This observation aligns with established risk factors for contrast media-induced nephropathy and other adverse effects, as individuals with preexisting vascular conditions may exhibit compromised renal function, thereby impairing the clearance of contrast agents[13]. Consequently, monitoring of these patients should be conducted with greater precision, particularly in assessing their renal function and cardiac status. If necessary, the dosage of contrast agent should be adjusted accordingly. Additionally, the observation that digestive system diseases, endocrine and metabolic disorders, and tumors rank third to fifth in terms of ADR frequency is consistent with the increasing application of ICM in imaging for oncological and metabolic conditions[14].

Furthermore, our clinical follow-up revealed that patients are frequently required to fast before examinations. For patients with a history of allergies, whether related or unrelated to ICM, no statistically significant difference was observed in the overall incidence of ADRs between the fasting and non-fasting groups. However, among patients with a history of non-ICM-related allergies, the incidence of severe ADRs was significantly greater in the fasting group than in the non-fasting group[15]. Numerous studies have highlighted that the traditional fasting policy lacks evidence-based justification and may exacerbate patient discomfort, such as hypoglycemia. Consequently, several medical institutions in Europe and China have progressively eliminated routine fasting requirements.

Most ADRs (71.14%) occurred on the same day that ICM was administered, which underscores the importance of immediate monitoring after contrast agent administration. However, notably, a significant proportion of reactions related to iodixanol and iomeprol occurred more than 24 h after administration. This delayed onset of adverse reactions may be indicative of a different immune-mediated response that requires further investigation, particularly in terms of monitoring protocols for patients receiving these agents[16].

In this study, rash emerged as the most prevalent adverse reaction, accounting for 54.76% of all reported adverse events, followed by itching, which accounted for 35.18%. These findings align with previous research, underscoring that dermatological symptoms are the predominant manifestations of adverse reactions associated with ICM [17, 18]. Skin reactions induced by ICM, such as rashes and itching, are typically immune-mediated allergic responses, which are particularly prevalent among patients with repeated exposure. Research indicates that non-IgE-mediated pseudoallergic reactions constitute the primary mechanism underlying skin symptoms in the majority of cases [19]. Consequently, the management of these dermatological symptoms should prioritize the patient’s allergy history and prior exposure to contrast agents. Furthermore, gastrointestinal adverse reactions, including nausea and vomiting, as well as respiratory adverse reactions, such as chest tightness, dyspnea, and throat constriction, are also observed with notable frequency [20]. These symptoms may be attributed to the chemical toxicity of the contrast agent and warrant significant clinical consideration.

Among the severe adverse reactions observed, rash and pruritus were the most prevalent symptoms. However, the incidence of anaphylactic shock, accounting for 9.69% of severe adverse reactions, warrants significant attention. This underscores the necessity for heightened vigilance regarding the risk of anaphylactic shock when iodinated contrast agents are administered and emphasizes the importance of having appropriate emergency preparedness measures in place [21].

Our investigation identified 128 novel severe ADRs associated with ICM, encompassing symptoms such as chest tightness, liver dysfunction, eyelid edema, and severe dermatological reactions. The occurrence of chest tightness, particularly when linked to the use of iodixanol and iohexol, may be indicative of cardiovascular stress or allergic responses, underscoring the importance of early detection and timely intervention, particularly in patients at elevated risk. Liver-related ADRs, including hepatocellular damage and abnormalities in liver function, underscore the potential hepatotoxic effects of ICM, especially in individuals with preexisting hepatic conditions. The presence of eyelid and periorbital edema suggests possible allergic reactions and warrants vigilant monitoring. Severe dermatological reactions, such as exfoliative dermatitis and generalized edema, imply more pronounced immune-mediated responses. Therefore, early diagnosis and the implementation of personalized monitoring strategies are essential for the effective management of these severe reactions, thereby ensuring patient safety, particularly among high-risk populations.

In this study, adverse reactions were observed across 17 distinct organ systems, with the highest incidence pertaining to skin and subcutaneous tissue disorders (69.62%), followed by systemic conditions and administration site reactions (7.39%) and gastrointestinal disorders (7.39%). These findings suggest that adverse reactions related to the ICM predominantly impact the integumentary and systemic systems, underscoring the necessity for clinical vigilance regarding symptoms and signs associated with these systems [22].

The management of ADRs associated with ICM predominantly involves symptomatic treatment. Corticosteroids, such as dexamethasone, are frequently utilized to address these reactions in conjunction with antihistamines such as loratadine, promethazine, and diphenhydramine, which are routinely administered for mild to moderate allergic responses. This therapeutic strategy aligns with contemporary clinical practices, wherein antihistamines and corticosteroids constitute the standard regimen for managing less severe ADRs [10, 23]. In instances of severe allergic reactions, such as anaphylaxis, more intensive interventions, including the administration of adrenaline (epinephrine), become necessary. This approach is consistent with established clinical guidelines for the management of life-threatening allergic reactions, highlighting the critical importance of timely and appropriate treatment to mitigate risks and ensure patient safety [21].

Skin testing, encompassing both skin prick tests (SPTs) and intradermal tests (IDTs), serves as a method to evaluate patients’ risk of allergy to iodine contrast agents, particularly in individuals with a prior history of allergic reactions [16, 24]. Nonetheless, the sensitivity of skin tests for iodine contrast agents is limited [25, 26]. Despite negative skin test results, 12.5% of patients may still experience allergic reactions upon re-exposure. Among those with immediate allergic reactions, only 8.1% are confirmed through skin testing, indicating a substantial false-negative rate [22]. In our study, 22 patients underwent skin testing for iodine contrast agents, with 13 receiving negative results. However, allergic reactions, including anaphylactic shock, occurred in these patients, further corroborating this limitation. Moreover, the skin testing procedure itself may provoke systemic allergic reactions. In our cases, one patient experienced anaphylactic shock as a result of the skin test, underscoring the necessity of conducting skin testing in facilities equipped for emergency management [27]. Consequently, ICM skin testing functions as a supplementary instrument for assessing allergy risk; however, its predictive value is constrained and should be evaluated alongside the patient’s clinical history [28]. Recent studies have indicated that, in patients deemed high risk, the integration of skin testing with a drug provocation test (DPT) may increase diagnostic precision [29].

This study offers significant insights into the epidemiology and clinical management of ICM-related ADRs; however, it is important to acknowledge several limitations. First, the study’s reliance on voluntary ADR reports may lead to underreporting or bias, particularly if less severe reactions are not documented. Second, the observational design of the study precludes the definitive establishment of causality, necessitating further prospective research to identify potential risk factors for severe reactions. Moreover, a more comprehensive analysis of the specific mechanisms underlying delayed reactions to iodixanol and iomeprol could enhance the understanding of the immunological pathways involved in these reactions.

## Conclusion

With the increasing prevalence of medical imaging and the growing number of individuals undergoing such procedures, concerns regarding adverse reactions to iodine-based contrast agents have become more prominent. Although this study utilized a descriptive analysis approach, it provides insights into the underlying disease risk, clinical manifestations, efficacy of various therapeutic interventions, and outcomes associated with adverse reactions to ICM to some extent. The potential risks associated with the iodine allergy test itself were also identified as requiring attention. This research helps increase clinicians’ awareness of the risks of ADRs to the ICM and offers foundational guidance on treatment options. Consequently, this study can inform improved management practices and enhance patient safety in the use of contrast agents.

This study is subject to several limitations. Owing to restrictions on system permissions, access to a comprehensive database necessary for determining total city usage was not possible. Consequently, the actual incidence of ADRs for each ICM could not be ascertained. The dataset used in this study was precleaned, limiting the availability of detailed medication information. For example, immediate hypersensitivity reactions occurring within one hour of ICM exposure could not be identified, as both the initiation time of medication and the onset of ADRs were recorded only daily. Additionally, as the data were derived from real-world sources, they are inherently subject to incompleteness and missing reports, which impose certain constraints on the study’s findings. Future efforts should focus on enhancing the monitoring and reporting of adverse reactions associated with ICM. It is imperative for pharmacists to bolster clinical education regarding ADRs, improve the monitoring of patient medication, and enhance the reporting, management, and analysis of ADR data. A prospective study examining the ADRs associated with ICM, which includes detailed patient medical histories and comorbidities, is necessary to facilitate the selection of more appropriate ICM for individual patients.

## Data Availability

The data that support the findings of this study are available from the corresponding author upon reasonable request.
